# Comparing the Pericapsular Nerve Group Block and the Lumbar Plexus Block for Hip Fracture Surgery: A Single-Center Randomized Double-Blinded Study

**DOI:** 10.3390/jcm13010122

**Published:** 2023-12-25

**Authors:** Tae Young Lee, Chan Jong Chung, Sang Yoong Park

**Affiliations:** Department of Anaesthesiology and Pain Medicine, College of Medicine, Dong-A University, Busan 49201, Republic of Korea; pinpd@dau.ac.kr (T.Y.L.); cjchung@dau.ac.kr (C.J.C.)

**Keywords:** analgesia, hip fracture surgery, lumbar plexus block, pericapsular nerve group block, regional analgesia

## Abstract

Lumbar plexus blocks (LPBs) are routinely employed for analgesia in hip fracture surgery; however, a novel regional technique, the pericapsular nerve group (PENG) block, potentially offers comparable pain reduction while preserving motor function. Patients aged 45–90 years who underwent hip fracture surgery were allocated to receive either a PENG block or an LPB for analgesia. The primary outcome was the incidence of quadriceps motor block (defined as the paresis or paralysis of the knee extension) at 12 h postoperatively. The secondary outcomes included the performance time, the time to first analgesic requirement, postoperative intravenous (IV) fentanyl consumption, the ability to undergo physiotherapy at 24 and 48 h, complications, sensory and motor block assessments, postoperative numeric rating scale (NRS) pain scores, and patient outcome questionnaires. There was a significantly lower incidence of quadriceps motor block at 6 h (26.7% vs. 80.0%; *p* < 0.001) and at 12 h (20.0% vs. 56.7%; *p* = 0.010). The PENG block provided better preservation of the sensory block as well as better performance time (*p* < 0.001) and time to first analgesia requirement (*p* = 0.034), whereas the LPB resulted in lower postoperative IV fentanyl consumption at 24 h (*p* = 0.013). The PENG block demonstrated superiority over the LPB in preserving quadriceps strength and patient satisfaction without any substantial complications, despite higher opioid consumption within the first 24 h post-surgery.

## 1. Introduction

According to a 2020 report, more than 1.5 million people experience hip fractures annually [1]. These fractures can result in substantial socioeconomic loss and a reduced quality of life [2,3]. While surgery is widely accepted as the standard treatment for hip fractures [4], it often leads to overwhelming postoperative pain [5], contributing to increased complications and delayed recovery [6]. The lumbar plexus block (LPB) is routinely employed for pain control in hip surgery. Many studies have shown that the LPB is superior to lumbar epidurals, a femoral block, a lateral quadratus lumborum block, and a suprainguinal fascia iliaca block (SIFIB) in terms of the time to the first analgesic requirement, postoperative opioid consumption, complications, and in the postoperative numeric rating scale (NRS) pain scores [7,8,9,10]. However, the LPB easily causes weakness of the quadriceps, which results in a delayed recovery and a later discharge date [7,8,9,10].

A promising alternative regional technique for postoperative analgesia after hip surgery is the pericapsular nerve group (PENG) block. The PENG block is an interfascial plane block that blocks the sensory branches including the articular branches of the femoral, the obturator, and the accessory obturator nerves to the anterior hip capsule, limiting weakness in the quadriceps or adductor muscles [11]. Several studies have indicated that the PENG block can offer sufficient analgesia and can improve quadriceps strength, with reduced requirements for opioids during postoperative care [12,13,14]. A comparative study with an SIFIB demonstrated excellent results in preserving quadriceps strength [15]; when compared with the group without the nerve block, the group with the PENG block showed no difference in quadriceps strength [16]. 

On the basis of these encouraging findings, we hypothesized that the PENG block may enable a better recovery than the LPB after hip fracture surgery. As direct comparisons between the PENG block and the LPB are lacking, we aimed to perform this comparison in hip fracture surgeries in this study, with the strength of the quadriceps muscle 12 h postoperatively as our primary outcome measure.

## 2. Methods

This randomized, double-blind, placebo-controlled study was conducted at a single institution and was approved by our hospital’s institutional review board. This study was registered in the Clinical Research Information Service at cris.nih.go.kr on 14 January 2022. All patients provided informed written consent prior to enrollment. This study was conducted in accordance with the principles of the Declaration of Helsinki and reported using the guidelines of the Consolidated Standards for Reporting Trials.

The participants comprised patients aged 45–90 years who underwent hip fracture surgery under general anesthesia at our hospital between January and October 2022, who had an American Society of Anesthesiologists physical status class of I, II, or III, and who agreed to receive a PENG block or an LPB. Exclusion criteria were infection in the area where the blocking would be performed; chronic opioid dependence; morbid obesity (a body mass index > 35 kg/m^2^); neurological deficits; a bleeding tendency; allergies to ropivacaine; poorly controlled diabetes; pregnancy; psychosis; and a lack of consent. Before surgery, the patients were randomly assigned to receive either a PENG block (the PENG block group) or an LPB (the LPB group). The total dose of ropivacaine (100 mg) was the same for both groups [15]. However, for the PENG blocks a concentration of 0.5 was used, in compliance with the 20 mL dose claimed by Girón-Arango et al. [11] In contrast, for the LPB group, a concentration of 0.25% in a 40 mL dose [9] was used. 

A computer-generated randomization was used to ensure a 1:1 ratio between the groups. A random assignment table was generated using nQuery Advisor^®^ version 7.0 (Statsols, BMDP Statistical Software Inc., Cork, Ireland), and the block sizes were randomly selected. We prepared sealed, numbered envelopes containing the group assignment information. A research assistant who was not involved in the anesthesia of the study participants opened the sealed envelope, confirmed each patient’s group assignment, and prepared 20 mL of 0.5% ropivacaine (for the PENG blocks) or 40 mL of 0.25% ropivacaine (for the LPBs). The anesthesiologists involved in the study were blinded to the group allocation, with one anesthesiologist performing the block and the other assessing its intraoperative and postoperative effects. The final investigator, who was aware of the allocation of groups but was not part of the block procedure, forwarded the complete data to a statistician. In contrast to the final investigator, all participants were blinded to their group allocation. The hip fracture surgery was performed by the same surgical team for all patients. Both groups received light sedation administered intravenously before surgery as a multimodal preemptive analgesia.

A standardized endotracheal general anesthesia was induced in all patients who subsequently received wound infiltration and a periarticular injection of 20 mL of 0.25% ropivacaine with ketorolac (30 mg) at the end of the surgical procedure to eliminate confounding factors related to the patients’ perception of superficial pain.

### 2.1. Performance of Nerve Blocks

The PENG block was performed with the patients in the supine position. A high-frequency linear ultrasound transducer (3–16 MHz; HS40 device; Samsung Medison Ultrasound, Seoul, Republic of Korea) was placed in the transverse section of the anterior superior iliac spine to identify the anterior inferior iliac spine, the iliopubic eminence, and the psoas tendon [11]. The in-plane technique was employed to advance the block needle laterally until it reached the space between the iliopubic eminence and the psoas tendon. After negative aspiration, 20 mL of 0.5% ropivacaine solution was used. The blocks were consistently performed by the same anesthesiologist, and the ultrasound screen was systematically moved away from the patient’s field of view during the procedure.

In the LPB group, the patients assumed the lateral decubitus position, with the surgical limb in the uppermost position. A local anesthetic (LA) (2–3 mL of 1% lidocaine) was infiltrated into the skin and subcutaneous tissue. An insulated nerve block needle (a 22-gauge Echoplex Plus needle; Vygon Vet. Ltd., London, UK) was inserted approximately 4 cm lateral to the midline in the paravertebral position under the guidance of a curvilinear ultrasound transducer (2–6 MHz; HS40 device; Samsung Medison Ultrasound, Seoul, Republic of Korea) [17]. The block needle was slowly advanced until either a needle–lumbar plexus contact was visualized or an ipsilateral quadriceps muscle contraction was elicited, indicating the needle tip’s proximity to the lumbar plexus. After a negative aspiration, 40 mL of 0.25% ropivacaine was injected at a divided dose over 1 min. Following the block procedure, the patients were returned to the supine position, and general anesthesia was induced surgery.

### 2.2. Postoperative Analgesic Regimen

For the postoperative pain management, all patients received standardized multimodal analgesics at 12 h intervals. Additionally, intravenous patient-controlled analgesia (IV-PCA) was prepared by combining 20 μg/kg of fentanyl and 0.6 mg of ramosetron in a saline solution. The baseline infusion rate, bolus required dose, and lockout time were 1 mL/h, 1 mL, and 10 min, respectively. All patients were trained to use the IV-PCA pump (Accufuser Plus M1015M; Woo Young Medical, Seoul, Republic of Korea) before surgery. The patients were connected to a pump after surgery, and their opioid consumption was assessed for 24 h after surgery. The patients were instructed to activate the pump only if their NRS pain score was 4 or higher. 

### 2.3. Primary Outcome

Our primary outcome measure, the strength of the quadriceps muscle on the operated side, was evaluated both before the PENG block administration and 12 h after surgery using a knee extension test in the supine position with the patients’ hips flexed to 45° and their knees flexed to 90°. The patients were instructed to first perform a knee extension against gravity and then against resistance. The knee extension was assessed on a three-point scale as follows: 0, no block; 1 = paresis (a decreased ability to extend the leg); and 2, paralysis (an inability to extend the leg) [9].

### 2.4. Secondary Outcomes

Sensory block assessments were conducted 6, 12, and 24 h after the administration of the block using a pinprick test. The jagged edges of a broken tongue depressor were applied to the skin over the lateral, anterior, and medial aspects of the mid-thigh. Sensory responses were evaluated using a three-point scale: 0 = no block, 1 = analgesia (the patient could feel touch but not cold), and 2 = anesthesia (the patient could not feel touch) [9]. The motor block was assessed by evaluating the knee extension and hip adduction. The knee extension was also assessed at 6 and 24 h using the same three-point scale as described for the primary outcome. To examine the hip adduction, the post-block strength was compared with a baseline strength at 6, 12, and 24 h. Before administering the PENG block or the LPB, a blood pressure cuff inflated to 40 mm Hg was placed between the knees of the patients. The patients were then instructed to squeeze the cuff as hard as possible and to sustain the effort. We defined the hip adduction scores of 0, 1, and 2 points as decreases in strength of 0–20%, 21–70%, and 71–90%, respectively, compared to the baseline measurement [9].

The secondary outcomes included the block performance time and the incidence of block-related adverse events (i.e., LA toxicity and dizziness). Postoperatively, the time to the first analgesic request, cumulative fentanyl consumption at 24 and 48 h, an inability to undergo physiotherapy at 24 and 48 h due to movement difficulties or pain, opioid-related side effects, in-hospital falls, and the time to readiness for discharge (defined as the time required to navigate stairs independently) were recorded.

At all postoperative time points (6, 12, 18, 24, and 48 h), patients were asked to indicate their perceived pain using a 0 to 10 NRS (0 = no pain; 10 = worst pain imaginable). The worst pain experienced during the previous 6 h was considered the maximum pain. 

Except for the performance time and the incidence of LA toxicity and dizziness, all other outcomes were assessed by a blinded investigator who also recorded demographic data and the duration of surgery.

Patient outcome questionnaires measured the sleep quality on the first night after the surgery using a consensus sleep diary [18] rated on a 0 to 4 point scale (4 = very good, 3 = good, 2 = fair, 1 = bad, and 0 = very bad), a satisfaction score (0 = worst, 100 = best), and a willingness to “go through the block again”.

### 2.5. Sample Size Calculation and Statistical Analysis

The sample size calculations were conducted using nQuery Advisor^®^ version 7.0 software (Statsols, BMDP Statistical Software Inc., Cork, Ireland). A retrospective survey of 100 patients after the LPB indicated an estimated 67% incidence of quadriceps motor block (paresis or paralysis) at 12 h. Our hypothesis was that we would observe a 20% decrease in the incidence among the PENG block group [15]. A calculated sample size of 25 patients per group was required to achieve a statistical power of 0.90 and a two-sided type I error of 0.05. Considering a dropout rate of 20%, a total sample size of 60 patients was deemed necessary.

The data are presented as the frequency and percentage for the categorical variables and as the median (minimum–maximum) or mean ± standard deviation for the numeric variables. Group differences in the study participants’ characteristics were compared using the appropriate statistical tests: the chi-squared test or Fisher’s exact test for the categorical variables and the independent *t*-test or Mann–Whitney U test for the continuous variables. The normality of the distribution was assessed using the Shapiro–Wilk test. Considering the nature of the repeated measurements, a generalized linear mixed model (GLMM) with random intercepts was employed. The GLMM included repeated measures of the numeric variables as dependent variables, group × time interactions as fixed effects, and the participants as random effects. To avoid assumptions regarding the covariance structure, an unstructured covariance matrix that varied across groups was utilized for the GLMM analysis. The post hoc analyses were performed using Bonferroni’s procedure.

All statistical analyses were conducted using an SPSS 26.0 (IBM Corp., Armonk, NY, USA), and *p*-values below 0.05 were considered statistically significant.

## 3. Results

**Figure 1:**
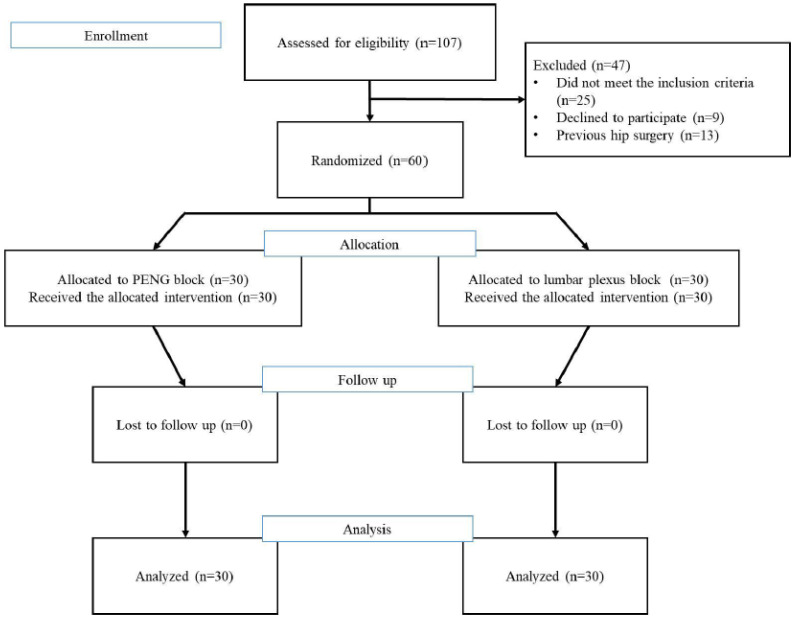
CONSORT diagram. Flow diagram of patients assessed using the protocol. PENG: pericapsular nerve block group.

During the study period, 107 patients were initially assessed for eligibility, of whom 60 met the inclusion criteria and agreed to participate. Patients were randomly assigned to two groups, and all 60 patients who completed the study were included in the final analysis (Figure 1). There were no clinically relevant differences in the demographics and preoperative characteristics between the groups (Table 1).

Compared with the PENG block group, the LPB group resulted in a higher incidence of quadriceps motor block at 6 and 12 h (80 vs. 26.7%, *p* < 0.001; and 56.7 vs. 20%, *p* = 0.010, respectively), as evidenced by impaired knee extension (Table 2). No intergroup differences were found in the hip adduction movements. Additionally, an LPB was associated with an increased sensory blockade of the anterior, lateral, and medial thighs at 6 and 12 h (all *p* < 0.001) compared to a PENG block (Table 2).

The PENG block demonstrated a superior performance and a shorter time to the first analgesic requirement (*p* < 0.001 and *p* = 0.034, respectively). Moreover, the PENG block group required a higher quantity of intravenous fentanyl within the first 24 h postoperatively (*p* = 0.013). However, there were no intergroup differences in terms of postoperative intravenous fentanyl consumption at 48 h, an inability to perform physiotherapy at 24 and 48 h owing to motor blockade or pain, the incidence of block-related adverse events, opioid-related side effects, in-hospital falls, or the time to discharge (Table 3). 

**Figure 2:**
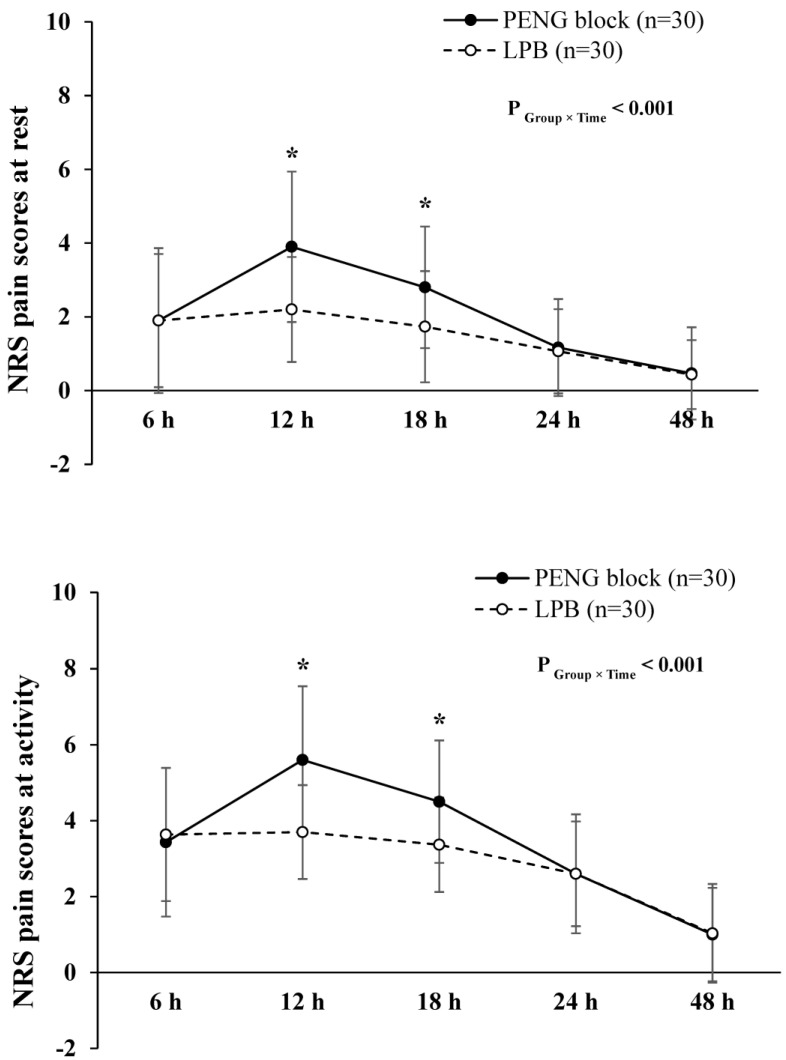
Postoperative pain score. Mean ± standard deviation (SD) plot of pain scores at rest and activity across the groups. *: A statistically significant difference. *p* values were derived from the Mann-Whitney U test and adjusted by the Bonferroni correction method. *p* _Group × Time_ values were derived from the Generalized Linear Mixed Model. The Shapiro-Wilk test was employed as the normality assumption test. NRS: numeric rating scale; PENG: pericapsular nerve block group; and LPB: lumbar plexus block group.

Two-way repeated measures analysis of variance demonstrated significant changes in the pain scores at rest and during activity (GLMM, *p* < 0.001), with a significant interaction between the group and the time (GLMM, *p* < 0.001). Post hoc analyses indicated that these differences stemmed from variations at 12 and 18 h (Figure 2).

Sleep quality, as assessed using a questionnaire, did not show any significant intergroup differences. However, the satisfaction scores were notably higher in the PENG block group (*p* < 0.001), and more participants expressed a positive inclination towards receiving the block again (*p* = 0.049) (Table 4).

## 4. Discussion

This randomized clinical trial compared the PENG block with the LPB in patients undergoing hip fracture surgery. The PENG block group demonstrated significantly better preservation of postoperative knee extension motor function at 6 and 12 h than the LPB group. These improvements allowed earlier mobilization after the hip fracture surgery and were associated with fewer complications, lower mortality, less pain, and shorter hospital stays [19,20,21]. Notably, no significant differences were observed between the groups in terms of performing physiotherapy. However, the ability of the PENG block to enhance exercise strength suggests improvements in physiotherapeutic performance. The absence of differences between the groups can be attributed to two factors. First, physiotherapeutic ability was considered a secondary variable in our study, potentially resulting in the test being underpowered to detect significant differences. Second and more importantly, the current postoperative protocols for hip fracture surgery may be inadequate to fully capitalize on the motion-sparing effects of the PENG block. Our findings validate the motor-sparing advantages of the PENG block over the LPB, although we must acknowledge that the knee extension and hip adduction rates were 6.7% and 26.7%, respectively. These results may be attributed to the spread of the LA to the femoral nerve [13]. Some authors have suggested that the placement of the needle tip inside the iliopubic eminence during the PENG block may impact obturator motor blockade [22], which can occur when a large amount of LA is injected [23]. Additionally, the high concentration of LA used in this trial may have caused residual sedation during recovery and difficulty in understanding the instructions. It is possible that this high concentration resulted in motor weaknesses, which warrant further investigation and which could explain the higher than expected impedance in knee extension motor strength.

Regarding the LA volume used in our study, the 20 mL administered for the PENG block reflects the volume recommended by Girón-Arango et al. [11]. A high-volume PENG block might not be an ideal option, and persistent postoperative pain may be partially attributed to the lack of an obturator nerve articular branch blockade [24]. In a 2015 LPB dose-finding trial, Sauter et al. [25] concluded that the minimum effective volume of 0.5% ropivacaine in 95% of patients (ED95) was 36 mL. We used 0.25% ropivacaine in 40ml for LPB is to use the same dose as 20ml 0.5% for PENG block. Interestingly, although the same overall dose of the LA was administered in both blocks, the results were not similar. A high volume and low concentrations did not extend the duration of analgesia compared to a low volume and high concentrations but did reduce the postoperative opioid requirement within 24 h [26]. The difference in the infusion volume and the dose between the two blocks may be a confounding factor in our results. However, the main goal of this study was not to compare two different volumes and doses of the LA, but the PENG block and the LPB. Additional studies are needed to define the success of the PENG block and the LPB and to elucidate the optimal dose, volume, and concentration of the LA.

The superior performance of the PENG block may be due to the patient’s posture during the procedure (a supine versus a lateral decubitus). The time to analgesic requirement indicates that the PENG block may result in shorter analgesia and reduced total intravenous fentanyl consumption over 24 h. We speculate that the reduced block duration is a result of the injected LA spreading around the pericapsule, causing a dispersion effect [24], and that the concentration of the LA in one large muscle affects the duration of the pain block [17]. Our results were limited to single-injection blocks and further studies are needed to confirm our findings regarding continuous PENG blocks and LPBs. 

In this study, a fundamental difference was observed in the area where the nerve block was performed. The PENG block targets the anterior hip capsule, whereas the LPB is administered at a location where the nerves gather centrally. Although the anterior capsule innervation is responsible for the majority of hip-related pain, the posterior capsule innervation, which is innervated by the quadratus femoris and superior gluteal nerves, is omitted. Both originate from the sacral plexus [27]. Further anatomical and clinical studies are needed to address this issue.

In terms of postoperative intravenous fentanyl consumption at 24 h, the standard deviation range (177–259 μg) was unexpectedly large, which could be attributed to the use of a posterior surgical approach. Given this variability in fentanyl consumption, it is possible that the sample size was insufficient to detect differences between the two study groups. 

Additionally, no adverse events directly related to the placement of the blocks were reported in either group. There was no observed difference in sleep quality; however, patient satisfaction and willingness to undergo the block again were significantly higher in the PENG block group (*p* < 0.001 and *p* = 0.049, respectively). 

## 5. Limitations

This study had several limitations. First, the participants may not have been fully blinded, since, although the ultrasound screen was turned away during the procedure, they could have deduced their group allocation as a result of their posture. Second, patients with hip fractures are older and have a higher incidence of dementia [28]. The strict patient selection criteria that excluded patients with cognitive impairment likely resulted in the exclusion of a significant number of patients, with selection bias as the result. Third, we did not differentiate between the range of motion and the “time to first walk.” Fourth, the power calculations were solely based on the primary outcome, and this study was not enabled to detect differences in fentanyl consumption and NRS scores. 

## 6. Conclusions

In conclusion, in patients undergoing hip fracture surgery under general anesthesia, a PENG block with 20 mL of 0.5% ropivacaine better preserved motor functions 6 h and 12 h postoperatively than an LPB. While the LPB group exhibited lower pain scores at rest and during activity at 12 h and 18 h postoperatively, no significant differences were observed in the pain scores, opioid consumption, and knee extension motor strength between patients receiving the PENG block and the LPB after 48 h. If the goal is to obtain benefits such as early rehabilitation by recovering motor function, the PENG block is recommended, but, if emphasis is placed on reducing pain or fentanyl consumption, the LPB is recommended considering the results a day after surgery. Additional mid- to long-term studies are required to clarify the advantages of postoperative clinical pathways, such as the identical volume and concentration of the LA afforded by these two blocks in patients with hip fractures.

## Figures and Tables

**Table 1:** Patients’ demographics and preoperative characteristics.

	Group		
Variable	PENG Block (*n* = 30)	LPB (*n* = 30)	*p*
Age (yr)	66.89 ± 8.26	69.09 ± 7.37	0.281 ^1^
Gender			0.592 ^3^
Male	10 (33.3)	12 (40.0)	
Female	20 (66.7)	18 (60.0)	
Body Mass Index (kg/m^2^)	24.09 ± 4.07	23.31 ± 3.94	0.308 ^2^
ASA Physical Status Classification			0.860 ^4^
1	4 (13.3)	3 (10.0)	
2	6 (20.0)	8 (26.7)	
3	20 (66.7)	19 (63.3)	
Surgical site			0.793 ^3^
Right	13 (43.3)	12 (40.0)	
Left	17 (56.7)	18 (60.0)	
Mobility			0.605 ^3^
Independent	15 (50.0)	17 (56.7)	
Assisted	15 (50.0)	13 (43.3)	
Chronic opiate usage			0.559 ^3^
Yes	9 (30.0)	7 (23.3)	
No	21 (70.0)	23 (76.7)	
Type of fracture			0.787 ^3^
Intracapsular	10 (33.3)	11 (36.7)	
Extracapsular	20 (66.7)	19 (63.3)	
Type of surgical repair			0.964 ^4^
Gamma nail	10 (33.3)	9 (30.0)	
Cannulated screw	6 (20.0)	5 (16.7)	
Hemiarthroplasty	10 (33.3)	12 (40.0)	
Total hip replacement	4 (13.3)	4 (13.3)	
Surgery time (min)	109.23 ± 20.44	112.01 ± 18.22	0.469 ^2^
Preoperative NRS pain score	8.43 ± 1.17	8.12 ± 1.93	0.663 ^2^

Values are either the frequency with the percentage in parentheses or the mean ± standard deviation. ^1^ *p* values were derived from the independent *t*-test. ^2^ *p* values were derived from the Mann–Whitney U test. ^3^ *p* values were derived using a chi-squared test. ^4^ *p* values were derived from Fisher’s exact test. The Shapiro–Wilk test was employed as the normality assumption test. ASA: American Society of Anesthesiologists; NRS: numeric rating scale; PENG: pericapsular nerve block group; and LPB: lumbar plexus block group.

**Table 2:** Sensory and motor block assessment.

	PENG Block			LPB			
Variable	No Block	Analgesia	Anesthesia	No Block	Analgesia	Anesthesia	*p*
Sensory block							
Lateral thigh							
at 6 h post-block	24 (80.0)	6 (20.0)	0 (0.0)	4 (13.3)	17 (56.7)	9 (30.0)	<0.001
at 12 h post-block	25 (83.3)	5 (16.7)	0 (0.0)	12 (40.0)	12 (40.0)	6 (20.0)	0.001
at 24 h post-block	27 (90.0)	3 (10.0)	0 (0.0)	24 (80.0)	6 (20.0)	0 (0.0)	0.472
Anterior thigh							
at 6 h post-block	25 (83.3)	5 (16.7)	0 (0.0)	2 (6.7)	22 (73.3)	6 (20.0)	<0.001
at 12 h post-block	27 (90.0)	3 (10.0)	0 (0.0)	12 (40.0)	14 (46.7)	4 (13.3)	<0.001
at 24 h post-block	30 (100.0)	0 (0.0)	0 (0.0)	26 (86.7)	4 (13.3)	0 (0.0)	0.112
Medial thigh							
at 6 h post-block	27 (90.0)	3 (10.0)	0 (0.0)	9 (30.0)	13 (43.3)	8 (26.7)	<0.001
at 12 h post-block	28 (93.3)	2 (6.7)	0 (0.0)	15 (50.0)	8 (26.7)	7 (23.3)	<0.001
at 24 h post-block	30 (100.0)	0 (0.0)	0 (0.0)	27 (90.0)	3 (10.0)	0 (0.0)	0.237
	PENG block			LPB			
Variable	No block	Paresis	Paralysis	No block	Paresis	Paralysis	*p*
Motor block							
Knee extension							
at 6 h post-block	22 (73.3)	6 (20.0)	2 (6.7)	6 (20.0)	21 (70.0)	3 (10.0)	<0.001
at 12 h post-block	24 (80.0)	5 (16.7)	1 (3.3)	13 (43.3)	15 (50.0)	2 (6.7)	0.010
at 24 h post-block	28 (93.3)	2 (6.7)	0 (0.0)	22 (73.3)	7 (23.3)	1 (3.3)	0.080
Hip adduction							
at 6 h post-bock	15 (50.0)	10 (33.3)	5 (16.7)	7 (23.3)	13 (43.3)	10 (33.3)	0.087
at 12 h post-bock	18 (60.0)	9 (30.0)	3 (10.0)	10 (33.3)	12 (40.0)	8 (26.7)	0.088
at 24 h post-bock	22 (73.3)	8 (26.7)	0 (0.0)	15 (50.0)	12 (40.0)	3 (10.0)	0.082

Values are the frequencies with the percentages in parentheses. *p* values were derived from Fisher’s exact test. PENG: pericapsular nerve block group; and LPB: lumbar plexus block group.

**Table 3:** Block performance data and postoperative outcomes.

	Group		
Variable	PENG Block (*n* = 30)	LPB (*n* = 30)	*p*
Performance time (min)	3.51 ± 0.84	5.22 ± 1.45	<0.001 ^2^
First analgesic required time (h)	8.45 ± 5.70	12.27 ± 6.73	0.034 ^2^
Postoperative intravenous fentanyl consumption at 24 h (μg)	495.79 ± 259.26	348.59 ± 177.54	0.013 ^1^
Postoperative intravenous fentanyl consumption at 48 h (μg)	762.13 ± 350.75	755.19 ± 275.81	0.932 ^1^
Inability to perform physiotherapy at 24 h due to motor blockade or pain, *n* (%)			1.000 ^3^
Yes	4 (13.3)	5 (16.7)	
No	26 (86.7)	25 (83.3)	
Inability to perform physiotherapy at 48 h due to motor blockade or pain, *n* (%)			1.000 ^3^
Yes	1 (3.3)	2 (6.7)	
No	29 (96.7)	28 (93.3)	
LA toxicity, *n* (%)			-
No	30 (100.0)	30 (100.0)	
Dizziness, *n* (%)			1.000 ^3^
Yes	2 (6.7)	2 (6.7)	
No	28 (93.3)	28 (93.3)	
PONV, *n* (%)			1.000 ^3^
Yes	3 (10.0)	3 (10.0)	
No	27 (90.0)	27 (90.0)	
Pruritis, *n* (%)			1.000 ^3^
Yes	2 (6.7)	1 (3.3)	
No	28 (93.3)	29 (96.7)	
Urinary retention, *n* (%)			-
No	30 (100.0)	30 (100.0)	
Respiratory depression, *n* (%)			-
No	30 (100.0)	30 (100.0)	
In-hospital falls, *n* (%)			0.492 ^3^
Fall as inpatient	0 (0.0)	2 (6.7)	
No fall recorded	30 (100.0)	28 (93.3)	
Time to readiness for discharge (days)	5.14 ± 1.27	5.44 ± 1.29	0.252 ^2^

Values are either the frequency with the percentage in parentheses or the mean ± standard deviation. ^1^ *p* values were derived from the independent *t*-test. ^2^ *p* values were derived from the Mann–Whitney U test. ^3^ *p* values were derived from Fisher’s exact test. The Shapiro–Wilk test was employed as the normality assumption test. LA: local anesthetic; PONV: postoperative nausea and vomiting; PENG: pericapsular nerve block group; and LPB: lumbar plexus block group.

**Table 4:** Patient outcome questionnaires.

	Group		
Variable	PENG Block (*n* = 30)	LPB (*n* = 30)	*p*
Quality of sleep (0~4)			0.246 ^2^
0	5 (16.7)	2 (6.7)	
1	9 (30.0)	5 (16.7)	
2	1 (3.3)	5 (16.7)	
3	3 (10.0)	5 (16.7)	
4	12 (40.0)	13 (43.3)	
Satisfaction score (0~100)	84.56 ± 3.77	72.95 ± 6.55	<0.001 ^1^
Would have the block again			0.049 ^2^
Yes	26 (86.7)	17 (56.7)	
No	3 (10.0)	8 (26.7)	
Ambivalent	1 (3.3)	5 (16.7)	

Values are either the frequency with the percentage in parentheses or the mean ± standard deviation. ^1^ *p* values were derived from the Mann-Whitney U test. ^2^ *p* values were derived from Fisher’s exact test. The Shapiro-Wilk test was employed as the normality assumption test. PENG: pericapsular nerve block group; and LPB: lumbar plexus block group.

## Data Availability

The data presented in this study are available upon reasonable request from the corresponding author.
